# Electronic and Hydrogen Storage Properties of Li-Terminated Linear Boron Chains Studied by TAO-DFT

**DOI:** 10.1038/s41598-018-31947-9

**Published:** 2018-09-10

**Authors:** Sonai Seenithurai, Jeng-Da Chai

**Affiliations:** 1 Department of Physics, National Taiwan University, Taipei, 10617 Taiwan; 2 Center for Theoretical Physics, National Taiwan University, Taipei, 10617 Taiwan; 3 Center for Quantum Science and Engineering, National Taiwan University, Taipei, 10617 Taiwan

## Abstract

It has been extremely difficult for conventional computational approaches to reliably predict the properties of multi-reference systems (i.e., systems possessing radical character) at the nanoscale. To resolve this, we employ thermally-assisted-occupation density functional theory (TAO-DFT) to predict the electronic and hydrogen storage properties of Li-terminated linear boron chains (Li_2_B_*n*_), with *n* boron atoms (*n* = 6, 8, …, and 16). From our TAO-DFT results, Li_2_B_*n*_, which possess radical character, can bind up to 4 H_2_ molecules per Li, with the binding energies in the desirable regime (between 20 and 40 kJ/mol per H_2_). The hydrogen gravimetric storage capacities of Li_2_B_*n*_ range from 7.9 to 17.0 wt%, achieving the ultimate goal of the United States Department of Energy. Accordingly, Li_2_B_*n*_ could be promising media for storing and releasing H_2_ at temperatures much higher than the boiling point of liquid nitrogen.

## Introduction

Hydrogen (H_2_) is a clean energy carrier, because only water vapor is emitted when converted into energy. Besides, hydrogen is quite plentiful on Earth in compound form (e.g., water (H_2_O)). Moreover, in terms of mass, the energy content of hydrogen is approximately three times that of gasoline. Hence, hydrogen can be a clean and green fuel, and has the potential to replace fossil fuels. Nonetheless, in terms of volume, the energy content of hydrogen is extremely low, when compared with that of gasoline. Therefore, efficient, economical, and safe hydrogen storage methods need to be developed for adopting hydrogen as a fuel in fuel cell vehicles^1–6^. The conventional high-pressure method where hydrogen is stored in carbon fiber reinforced plastic (CFRP) tanks at rather high pressures (e.g., between 350 and 700 bar) and the cryogenic method where hydrogen is stored at temperatures below the boiling point of H_2_ (about 20 K) are both unsuitable for onboard vehicle applications, due to the safety issues and high energy costs, respectively. Accordingly, it remains very difficult to efficiently store hydrogen in a lightweight and safe container^6^.

Presently, metal-organic frameworks (MOFs) and metal hydrides are adopted for storing hydrogen. As far as MOFs are concerned, the hydrogen storage capacities can be large due to the pore structure and high surface area of MOFs. Nevertheless, the hydrogen desorption temperatures for MOFs are rather low. On the other hand, in spite of their large hydrogen storage capacities, the hydrogen desorption temperatures for metal hydrides (e.g., MgH_2_, AlH_3_, LiBH_4_, and NaAlH_4_) can be very high, and the kinetics can be very slow (due to the formation/breaking of covalent and/or ionic bonds during the adsorption/desorption of hydrogen). Based on simple thermodynamic arguments, the hydrogen binding energy on a hydrogen storage material (HSM) has to lie between 20 and 40 kJ/mol per H_2_, for hydrogen uptake and release at near-ambient conditions^7–9^. However, among existing MOFs and metal hydrides, none can satisfy all the required conditions in order to use as an efficient HSM in onboard vehicles. Furthermore, on the basis of the ultimate goal of the United States Department of Energy (USDOE), a hydrogen gravimetric storage capacity of 6.5 wt% is required for a driving range of about 500 km^6^. Consequently, finding a HSM with all desirable properties has been very challenging.

Recently, carbon nanostructures have emerged as the potential materials for technological applications. The flexible bond formation (sp^1^, sp^2^, and sp^3^ hybridization) of carbon yields a very wide range of nanostructures which possess unique properties^10^. These nanostructures have been the test ground for studying many exotic phenomena. Especially, the discovery of C_60_ fullerene and the one-dimensional (1D) carbon nanotubes has revealed the potential of nanomaterial applications in diverse fields. Later, the discovery of graphene, the first ever two-dimensional (2D) material, has unlocked new possibilities in nanoscience and nanotechnology. This yields other 2D and quasi-2D materials, such as silicene, phosphorene, boron nitride nanomaterials, transition-metal dichalcogenides, single layers of metal oxides, and very recently, boron nanomaterials^11,12^. Since graphene is a semimetal or zero-gap semiconductor, its applications in electronics are impossible, unless a band gap can be opened by means of doping, defect formation, functionalization, and so on. Therefore, searching for other nanomaterials with better properties than graphene is in full swing.

In this pursuit, boron nanostructures are currently under intensive investigation to explore their electronic properties and potential applications. Due to recent advances in theoretical methods and experimental techniques, several boron nanostructures have been predicted and/or synthesized, and some of their basic properties and potential applications have been reported^13–21^. The observation of the Dirac cone^22^ in *β*_12_ boron sheet grown on Ag(111) has increased interest in these boron nanomaterials, due to their potential applications in electronics and possible exotic properties. The clusters ${{\rm{B}}}_{13}^{+}$ and ${{\rm{B}}}_{19}^{-}$ exhibit fluxional behavior, which has the potential for molecular Wankel motors^15^. Theoretical predictions have shown that B_40_ is a potential anode material for Li-ion battery applications^23^. Apart from their interesting electronic properties, these boron nanostructures can potentially be promising HSMs, because of their lightweight and high surface area. However, as carbon nanostructures are known to bind H_2_ molecules very weakly with the hydrogen binding energies typically less than 10 kJ/mol per H_2_ (primarily due to van der Waals (vdW) interactions), it is likely that most boron nanostructures also bind H_2_ molecules with insufficient binding energies. To increase the hydrogen binding energy to the desirable regime (between 20 and 40 kJ/mol per H_2_), the boron nanostructures can be suitably decorated/functionalized with some selective atoms (e.g., Li, Al, Ca, light transition metals, etc.)^2^.

However, transition metals are highly prone to clustering, and hence, the hydrogen storage capacities can easily decrease. Also, the first few H_2_ molecules can be adsorbed dissociatively (i.e., undesirable for applications at ambient conditions)^24^. Therefore, the dopant or decorating atoms should be rationally chosen with the following characteristics: a) they should be lightweight, b) they should not form clusters, and c) they can bind hydrogen molecularly. The element lithium (Li) seems to be ideal, as it can easily satisfy these conditions. When adsorbed or decorated, the 2*s* electron from the Li atom can be transferred to nanostructures (due to the difference between their electronegativity values), and hence, the Li atom can become a cation (i.e., a positively charged ion). The electric field from the dipole that is produced by the charge transfer is capable of polarizing the incoming H_2_ molecules (around the Li), and binding the H_2_ molecularly with the aforementioned desirable regime. This mechanism is referred to as charge-transfer induced polarization^2,25,26^. Therefore, Li-modified boron nanomaterials can potentially be HSMs.

**Figure 1:**
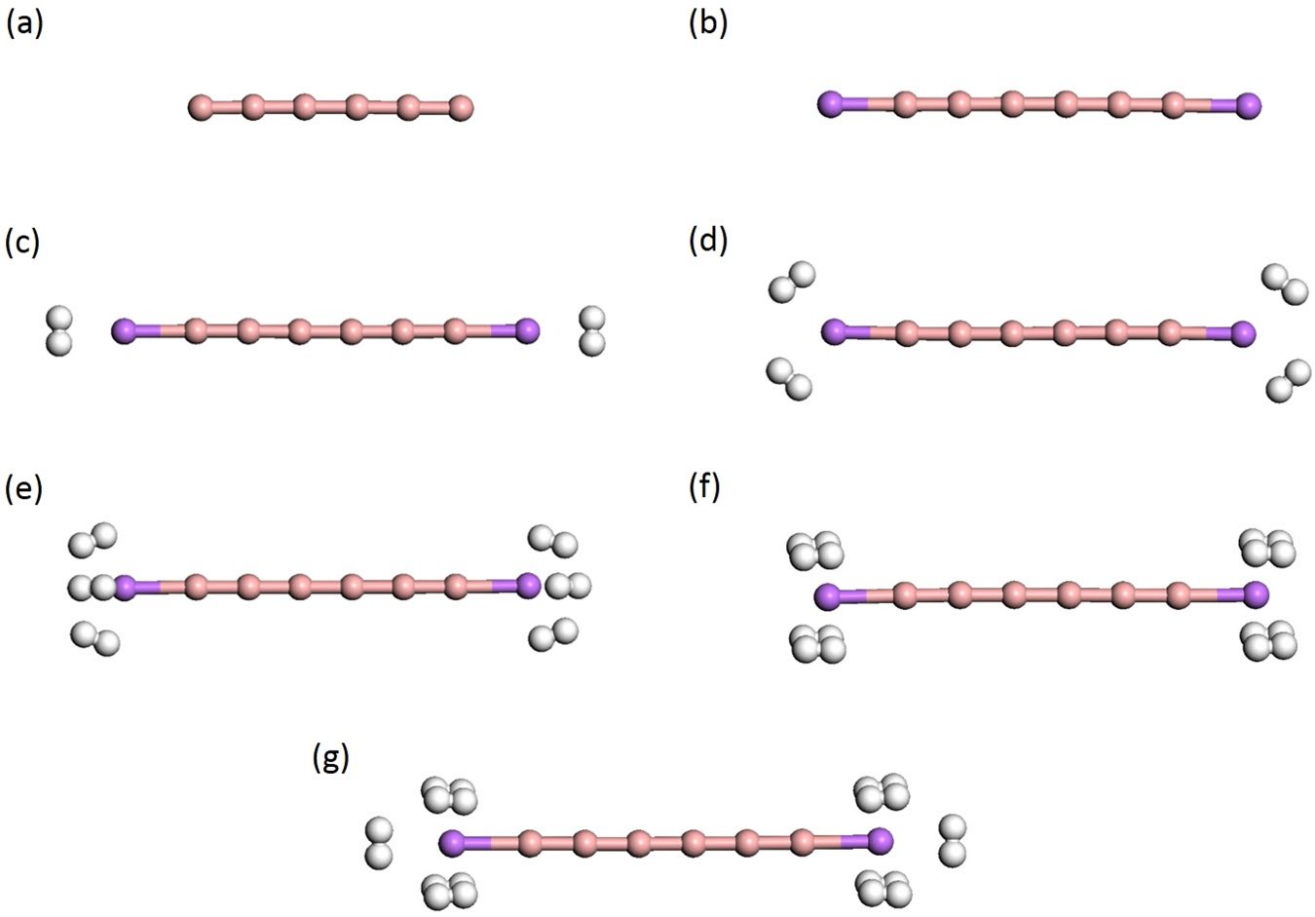
Structures of (**a**) linear boron chain (B_6_), (**b**) Li-terminated linear boron chain (Li_2_B_6_), and (**c**–**g**) Li_2_B_6_ with *x* H_2_ molecules (*x* = 1–5) adsorbed on each Li, obtained with TAO-BLYP-D. Here, pink, purple, and white balls represent B, Li, and H atoms, respectively. For the longer Li_2_B_*n*_ (*n* = 8, 10, …, and 16), the H_2_ adsorption patterns remain similar.

Among boron materials, there has recently been considerable interest in linear boron chains (B_*n*_), containing *n* boron atoms bonded with sp^1^ hybridization (see Fig. 1(a)), because of their promising electronic and mechanochemical properties. Their mechanochemistry studies have revealed that, under tension, boron atoms can form linear chains^27^. These boron chains show an interesting reversible structural phase transition between linear two-atom-wide narrow ribbons and single-atom chains under tension. The chains and narrow ribbons are linked by a tension-driven transformation and reported to be the stable structures. Understandably, linear boron chains can potentially be HSMs, if the chains are terminated with Li atoms. Note that Li-terminated linear boron chains (Li_2_B_*n*_) can be promising HSMs (see Fig. 1(b–g)), as they are lightweight materials associated with the aforementioned polarization mechanism^2,25,26^. However, it remains very challenging to synthesize Li_2_B_*n*_, as they can possess radical character (prevalent in low-dimensional systems because of quantum confinement effect^28^). Therefore, predicting the electronic and hydrogen storage properties of Li_2_B_*n*_ could pave the way for the progress in this field, and also play an important role in selecting ideal materials for nanoelectronics and optoelectronics applications.

Currently, electronic structure calculations on systems at the nanoscale are mainly performed using Kohn-Sham density functional theory (KS-DFT)^29^ with approximate exchange-correlation (XC) density functionals^30^. However, KS-DFT with traditional XC density functionals, such as BLYP-based (e.g., BLYP^31,32^, B3LYP^33,34^, and B2-PLYP^35^), PBE-based (e.g., PBE^36^, PBE0^37^, PBE0-2^38^, PBE0-DH^39^, and PBE-QIDH^40^), and *ω*B97-based (e.g., *ω*B97^41^, *ω*B97X^41^, *ω*B97X-D3^42^, and *ω*B97X-2^43^) functionals, may not be reliable in predicting the properties of multi-reference systems (i.e., systems possessing radical character), wherein *ab initio* multi-reference electronic structure methods, such as the density matrix renormalization group (DMRG) approach and multi-reference configuration interaction (MRCI) methods, are usually required^44,45^. Despite their high predictive accuracy, calculations based on *ab initio* multi-reference electronic structure methods can however be computationally infeasible for systems at the nanoscale (particularly for geometry relaxation). Consequently, the study of multi-reference systems at the nanoscale remains extremely difficult for conventional computational approaches.

Aiming to achieve a decent balance between accuracy and efficiency for the study of multi-reference systems at the nanoscale, thermally-assisted-occupation density functional theory (TAO-DFT)^46^ and its extensions^47–49^ have recently been proposed. On the basis of the physical arguments given in Section III.E of ref.^46^ and the numerical investigations presented in Section IV of ref.^46^, the static correlation energy of a system can be properly described by the entropy contribution (i.e., a function of the fictitious temperature and orbital occupation numbers (an implicit density functional)), even when a local or semilocal XC density functional is employed in TAO-DFT. Similar to the static correlation energy of a system, the entropy contribution in TAO-DFT is always nonpositive, yielding insignificant contributions for a single-reference system, and significantly lowering the total energy of a multi-reference system. Note that the inclusion of fractional occupation numbers in electronic structure calculations has been recently explored in some directions. For example, the fractional occupation number weighted electron density (FOD) analysis has been recently developed for a real-space measure and visualization of static correlation effects^50,51^, yielding promising applications to carbon nanoforms^52^.

Note that TAO-DFT is similar to KS-DFT in computational efficiency. Moreover, TAO-DFT reduces to KS-DFT when the static correlation energy of a system is insignificant, enabling a well-balanced description for both systems possessing non-radical character and systems possessing radical character^53–57^. In our previous TAO-DFT studies, Li-adsorbed acenes^55^ and Li-terminated linear carbon chains (Li_2_C_*n*_)^57^ were found to be promising HSMs at near-ambient conditions, showing that the search for promising HSMs can be extended to large systems possessing radical character. Although Li_2_C_*n*_ and Li_2_B_*n*_ look similar in structure, their electronic and hydrogen storage properties are distinctly different. In particular, Li_2_C_*n*_ were found to exhibit oscillatory diradical behavior with increasing chain length^57^, while Li_2_B_*n*_ exhibit increasing polyradical character with the increase of chain length (as will be seen below). Owing to its reasonable accuracy in predicting the properties of multi-reference systems at the nanoscale, we employ TAO-DFT to predict the electronic and hydrogen storage properties of Li_2_B_*n*_ (*n* = 6, 8, …, and 16) in the present study.

## Computational Details

We perform all calculations with Q-Chem 4.4^58^. Results are obtained from TAO-BLYP-D^47^ (i.e., TAO-DFT employing the Becke-Lee-Yang-Parr XC density functional with dispersion corrections (BLYP-D)^59^ and the *θ*-dependent density functional based on the local density approximation (LDA)) with the fictitious temperature *θ* = 7 mhartree^46,47^, using the 6–31 G(d) basis set and the numerical grid containing 75 radial points in the Euler-Maclaurin quadrature and 302 angular points in the Lebedev grid.

## Results and Discussion

### Electronic Properties

**Figure 2:**
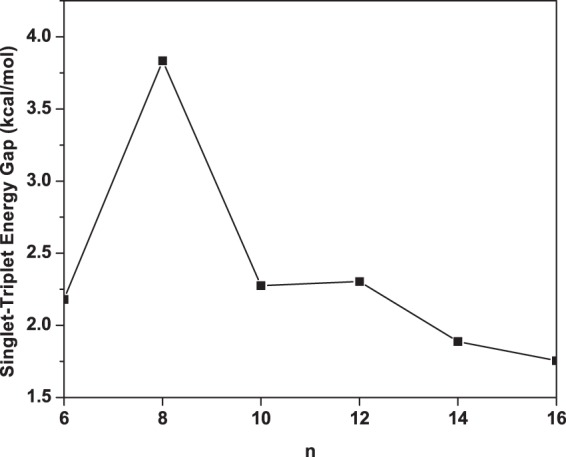
Singlet-triplet energy gap of Li_2_B_*n*_, obtained with TAO-BLYP-D.

To begin with, we perform spin-unrestricted TAO-BLYP-D calculations to obtain the lowest singlet and lowest triplet states of Li_2_B_*n*_ (*n* = 6, 8, …, and 16), with the respective geometries being fully relaxed^57^. Subsequently, we calculate the singlet-triplet energy gap of Li_2_B_*n*_ as

1 $${E}_{{\rm{ST}}}={E}_{{\rm{T}}}-{E}_{{\rm{S}}},$$

where *E*_S_ and *E*_T_ are the lowest singlet and lowest triplet energies, respectively, of Li_2_B_*n*_. As presented in Fig. 2, Li_2_B_*n*_ (*n* = 6, 8, …, and 16) has a singlet ground state (i.e., similar to Li_2_C_*n*_^57^). As *n* increases, *E*_ST_ changes drastically, implying that the electronic properties of Li_2_B_*n*_ can be properly tuned by changing the length of Li_2_B_*n*_.

For the exact theory, the lowest singlet energies of Li_2_B_*n*_ obtained with spin-restricted and spin-unrestricted calculations should be identical, due to the symmetry constraint^46–48,60^. To see if this property remains valid here, spin-restricted TAO-BLYP-D calculations are additionally performed for the lowest singlet energies on the corresponding optimized geometries. It is found that the lowest singlet energies of Li_2_B_*n*_ obtained with spin-restricted and spin-unrestricted TAO-BLYP-D calculations are numerically identical, indicating that our spin-unrestricted TAO-BLYP-D calculations do not yield unphysical symmetry-breaking solutions.

**Figure 3:**
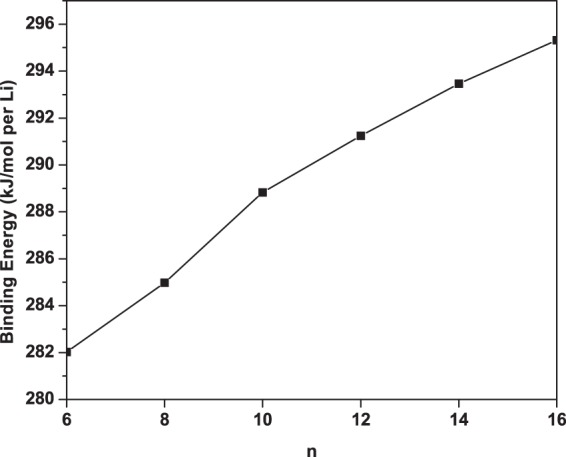
Li binding energy on B_*n*_, obtained with TAO-BLYP-D.

Strong binding of terminating Li atoms in Li_2_B_*n*_ is essential for reversible hydrogen storage applications. In order to know if the terminating Li atoms are stable, we calculate the Li binding energy on B_*n*_ using^57^

2 $${E}_{b}({\rm{Li}})=({E}_{{{\rm{B}}}_{n}}+2{E}_{{\rm{Li}}}-{E}_{{{\rm{Li}}}_{2}{{\rm{B}}}_{n}}\mathrm{)/2},$$

where ${E}_{{{\rm{B}}}_{n}}$, *E*_Li_, and ${E}_{{{\rm{Li}}}_{2}{{\rm{B}}}_{n}}$ are the total energies of B_*n*_, Li, and Li_2_B_*n*_, respectively. Subsequently, the standard counterpoise method^61^ is employed to correct the basis set superposition error (BSSE) associated with *E*_*b*_(Li). As can be seen in Fig. 3, the Li atoms can strongly bind with the B_*n*_ chain (and form Li_2_B_*n*_) with binding energies ranging from 282 to 295 kJ/mol per Li. Such high binding energies are desirable for reversible applications, as the dopant atoms should remain bound to B_*n*_ during the desorption of hydrogen molecules. The bonding of Li to B_*n*_ should be ionic due to the electronic charge transfer from Li to B_*n*_, which is expected to enhance the H_2_ adsorption to the Li atoms (as will be shown and discussed later).

**Figure 4:**
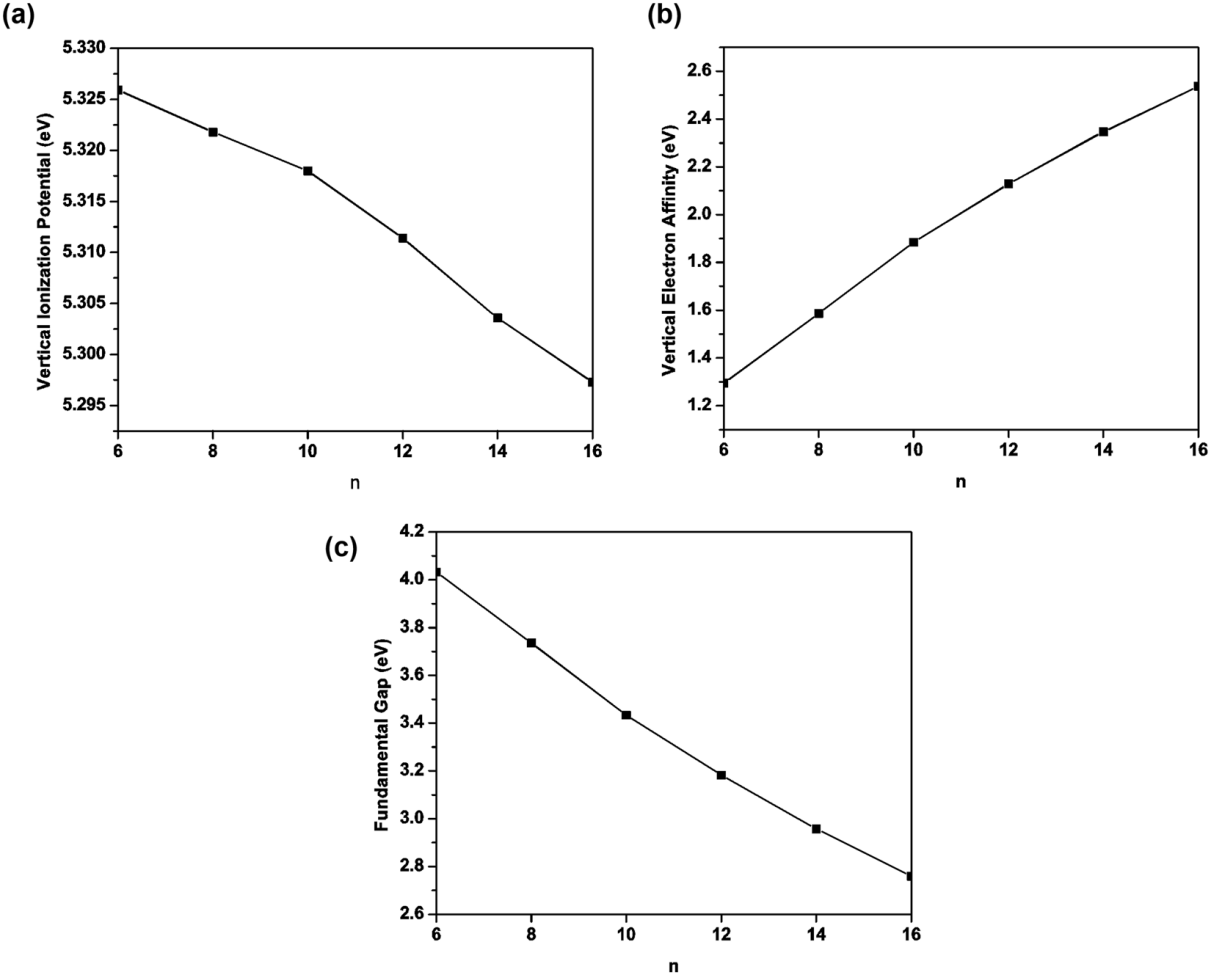
(**a**) Vertical ionization potential, (**b**) vertical electron affinity, and (**c**) fundamental gap for the ground state of Li_2_B_*n*_, obtained with TAO-BLYP-D.

The possibility of Li_2_B_*n*_ for photovoltaic applications is assessed here. At the optimized geometry of the lowest singlet state (i.e., the ground state) of Li_2_B_*n*_, spin-unrestricted TAO-BLYP-D is employed to calculate the vertical ionization potential (i.e., the energy difference between the cationic and neutral charge states)

3 $${{\rm{IP}}}_{v}={E}_{tot}({\rm{cation}})-{E}_{tot}\,({\rm{neutral}}),$$

vertical electron affinity (i.e., the energy difference between the neutral and anionic charge states)

4 $${{\rm{EA}}}_{v}={E}_{tot}({\rm{neutral}})-{E}_{tot}\,({\rm{anion}}),$$

and fundamental gap

5 $${E}_{g}={{\rm{IP}}}_{v}-{{\rm{EA}}}_{v},$$

via the Δ self-consistent field (ΔSCF) approach. As the chain length of Li_2_B_*n*_ increases, IP_*v*_ monotonically decreases, and EA_*v*_ monotonically increases, yielding a monotonically decreasing *E*_*g*_ (see Fig. 4). The IP_*v*_ value is found to be less sensitive to the chain length of Li_2_B_*n*_ than the EA_*v*_ and *E*_*g*_ values. Note also that the *E*_*g*_ value of Li_2_B_*n*_ (*n* = 14 and 16) is within the most interesting range (1 to 3 eV), giving promise for applications of Li_2_B_*n*_ in nanophotonics. Note that our theoretical results may guide further experimental studies on Li_2_B_*n*_.

**Figure 5:**
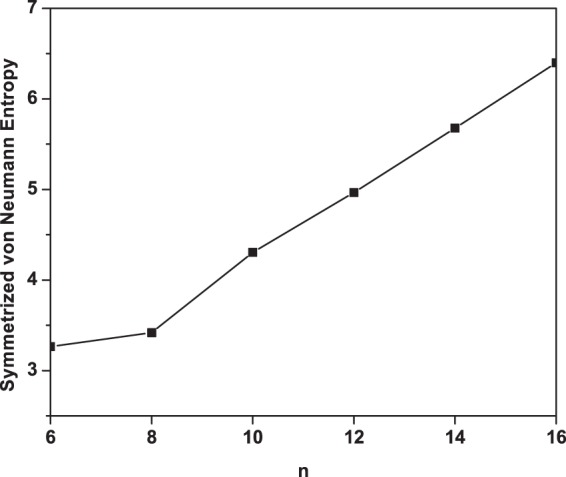
Symmetrized von Neumann entropy for the ground state of Li_2_B_*n*_, obtained with TAO-BLYP-D.

Here, we assess the multi-reference character of Li_2_B_*n*_ by calculating the symmetrized von Neumann entropy^47,48,53,55–57,60^

6 $${S}_{{\rm{vN}}}=-\,\frac{1}{2}\,\sum _{i=1}^{\infty }\,\{{f}_{i}\,\mathrm{ln}({f}_{i})+\mathrm{(1}-{f}_{i})\,\mathrm{ln}\,\mathrm{(1}-{f}_{i})\}$$

for the ground state of Li_2_B_*n*_. In Eq. (6), the occupation number of the *i*^th^ orbital calculated by TAO-BLYP-D (denoted as *f*_*i*_), which takes a value between zero and one, is close to the *i*^th^ natural orbital occupation number^46–48^. For a single-reference system ({*f*_*i*_} are approximately equal to either zero or one), *S*_vN_ is negligible. However, for a multi-reference system ({*f*_*i*_} are distinctly different from either zero or one for active orbitals, and are approximately equal to either zero or one for other orbitals), *S*_vN_ raises with the number of fractionally occupied orbitals (i.e., active orbitals). As presented in Fig. 5, *S*_vN_ increases with the chain length of Li_2_B_*n*_, implying that the multi-reference character of Li_2_B_*n*_ should generally increase with the chain length.

**Figure 6:**
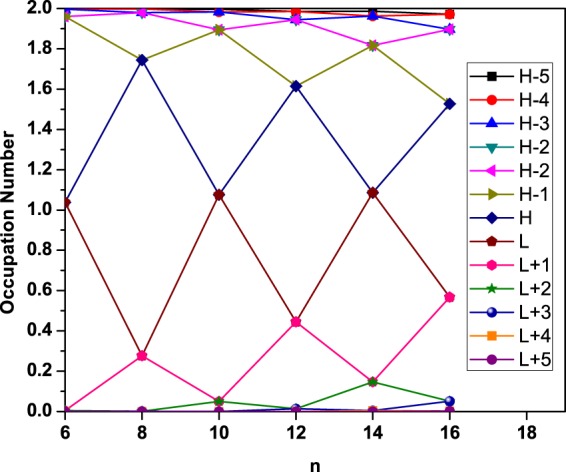
Active orbital occupation numbers (H−5, …, H−1, H, L, L+1, …, and L+5) for the ground state of Li_2_B_*n*_, obtained with TAO-BLYP-D. For brevity, HOMO and LUMO are denoted as H and L, respectively.

To further illustrate the reasons of the increase of *S*_vN_ with *n*, the active orbital occupation numbers for the ground state of Li_2_B_*n*_, obtained with TAO-BLYP-D, are plotted in Fig. 6. For Li_2_B_*n*_ (containing *N* electrons), the highest occupied molecular orbital (HOMO) is given by the (*N*/2)^th^ orbital, and the lowest unoccupied molecular orbital (LUMO) is given by the (*N*/2 + 1)^th^ orbital^46,48,53,56,57^. As shown, the number of fractionally occupied orbitals oscillatorily increases with the chain length of Li_2_B_*n*_, implying that the multi-reference character of Li_2_B_*n*_ should generally increase with *n* (see Table S1 in Supplementary Information).

Based on the above results, the longer Li_2_B_*n*_, which have the smaller *E*_ST_ values, smaller *E*_*g*_ values, larger *S*_vN_ values, and more significant polyradical character, are expected to possess stronger static correlation effects than the shorter Li_2_B_*n*_. Since KS-DFT employing traditional XC density functionals cannot reliably predict the properties of systems possessing radical character, and calculations based on *ab initio* multi-reference electronic structure methods are computationally infeasible for systems at the nanoscale (e.g., the longer Li_2_B_*n*_), it is well justified to employ TAO-DFT in the present study.

### Hydrogen Storage Properties

To begin with, we first examine the potential of B_*n*_ for hydrogen storage applications. Our preliminary TAO-BLYP-D results show that B_*n*_ can only adsorb H_2_ molecules with very weak binding energies (i.e., less than 5 kJ/mol per H_2_), mainly governed by vdW interactions. Therefore, B_*n*_ can be useful for hydrogen storage only at very low temperatures. Besides, B_*n*_ can only bind very few H_2_ molecules, since the interactions between the adsorbed H_2_ molecules at short separation distances are repulsive. Therefore, the average hydrogen binding energy on B_*n*_ should decrease, as the number of the adsorbed H_2_ molecules increases. Accordingly, B_*n*_ should be modified to realize a promising HSM at ambient conditions.

**Figure 7:**
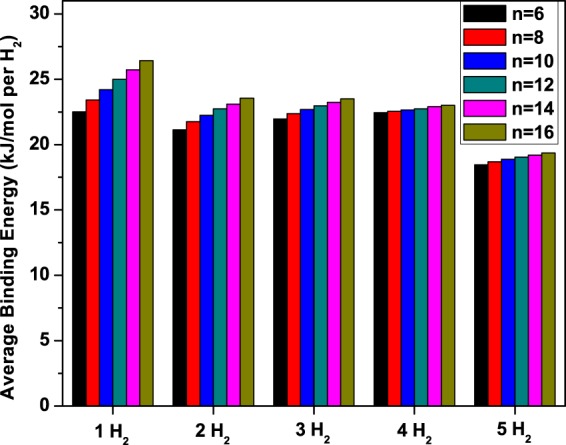
Average hydrogen binding energy on Li_2_B_*n*_ (*n* = 6, 8, …, and 16) with *x* H_2_ molecules (*x* = 1–5) adsorbed on each Li, obtained with TAO-BLYP-D.

In the following, the hydrogen storage properties of Li_2_B_*n*_ (*n* = 6, 8, …, and 16) are studied using TAO-BLYP-D. At the optimized geometry of the lowest singlet state (i.e., the ground state) of Li_2_B_*n*_, we initially put *x* H_2_ molecules (*x* = 1–5) at several locations on the chain, and subsequently optimize the structures to get the most stable geometry. However, it is found that the H_2_ molecules are adsorbed at the Li sites. All the H_2_ molecules can be adsorbed molecularly to the Li atoms, and this molecular adsorption is preferable for practical hydrogen storage applications. Here, we calculate the average hydrogen binding energy on Li_2_B_*n*_ using^57^

7 $${E}_{b}({{\rm{H}}}_{2})=({E}_{{{\rm{Li}}}_{2}{{\rm{B}}}_{n}}+2x{E}_{{{\rm{H}}}_{2}}-{E}_{{{\rm{Li}}}_{2}{{\rm{B}}}_{n}-2x{{\rm{H}}}_{2}}\mathrm{)/(2}x),$$

where ${E}_{{{\rm{H}}}_{2}}$, ${E}_{{{\rm{Li}}}_{2}{{\rm{B}}}_{n}}$, and ${E}_{{{\rm{Li}}}_{2}{{\rm{B}}}_{n}-2x{{\rm{H}}}_{2}}$ are the total energies of H_2_, Li_2_B_*n*_, and Li_2_B_*n*_ with *x* H_2_ molecules adsorbed on each Li, respectively. Necessarily, to account for BSSE, the aforementioned counterpoise method is used^61^. The BSSE associated with *E*_*b*_(H_2_) is estimated to range from 2.16 to 2.69 kJ/mol per H_2_ for *x* = 1–5 (see Tables S2 and S3 in Supplementary Information). There is a significant error due to BSSE, which denotes the importance of BSSE correction in H_2_ adsorption binding energy calculations. As presented in Fig. 7, *E*_*b*_(H_2_) ranges from 21 to 26 kJ/mol per H_2_ for *x* = 1–4, and ranges from 18 to 19 kJ/mol per H_2_ for *x* = 5, lying in (or very close to) the aforementioned desirable binding energy regime (between 20 and 40 kJ/mol per H_2_).

Besides the average hydrogen binding energy, the successive hydrogen binding energy should also be computed to assess the actual hydrogen storage capacity. Here, we calculate the successive hydrogen binding energy on Li_2_B_*n*_ using^57^

8 $${E}_{b,y}({{\rm{H}}}_{2})=({E}_{{{\rm{Li}}}_{2}{{\rm{B}}}_{n}-\mathrm{2(}y-\mathrm{1)}{{\rm{H}}}_{2}}+2{E}_{{{\rm{H}}}_{2}}-{E}_{{{\rm{Li}}}_{2}{{\rm{B}}}_{n}-2y{{\rm{H}}}_{2}}\mathrm{)/2.}$$

**Figure 8:**
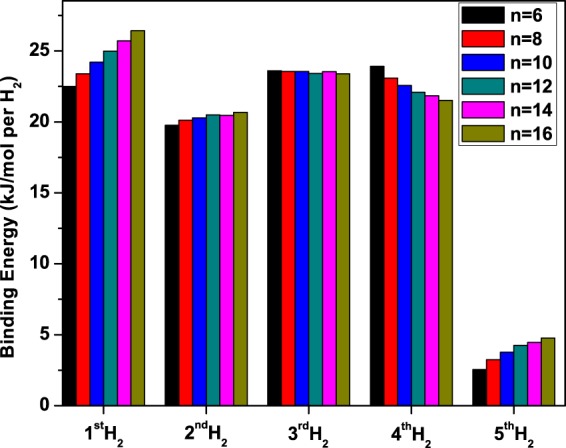
Binding energy of the *y*^th^ H_2_ molecule (*y* = 1–5) on Li_2_B_*n*_ (*n* = 6, 8, …, and 16), obtained with TAO-BLYP-D.

Here, *E*_*b*,*y*_(H_2_) is the binding energy of the *y*^th^ H_2_ molecule (*y* = 1–5) on Li_2_B_*n*_. Here also, the aforementioned counterpoise method^61^ is adopted to correct the BSSE associated with the binding energies. The BSSE associated with *E*_*b*,*y*_(H_2_) is estimated to range from 1.39 to 3.18 kJ/mol per H_2_ for *y* = 1–5 (see Tables S4 and S5 in Supplementary Information). There is a significant error due to BSSE, which also denotes the importance of BSSE correction in such studies. As shown in Fig. 8, *E*_*b*,*y*_(H_2_) ranges from 20 to 26 kJ/mol per H_2_ for *y* = 1–4, and ranges from 3 to 5 kJ/mol per H_2_ for *y* = 5. This denotes that only the first four H_2_ molecules (on each Li) are adsorbed in the desirable binding energy regime, and the fifth H_2_ molecule is adsorbed weakly (possibly due to vdW interactions and this is useful for hydrogen storage only at ultra low temperatures).

**Figure 9:**
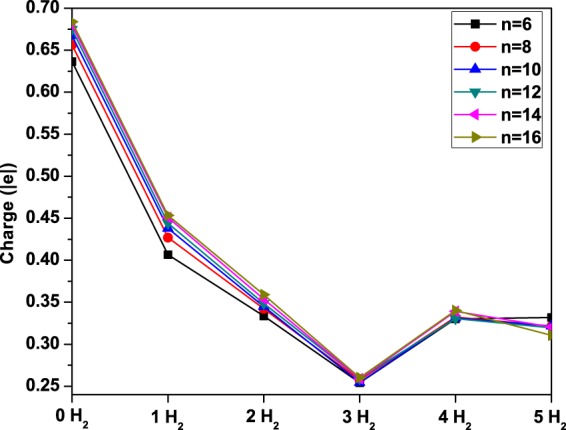
Li atomic charge for Li_2_B_*n*_ (*n* = 6, 8, …, and 16) with *x* H_2_ molecules (*x* = 0–5) adsorbed on each Li, obtained with TAO-BLYP-D. Here, the CHELPG scheme is employed to calculate the Li atomic charge.

**Figure 10:**
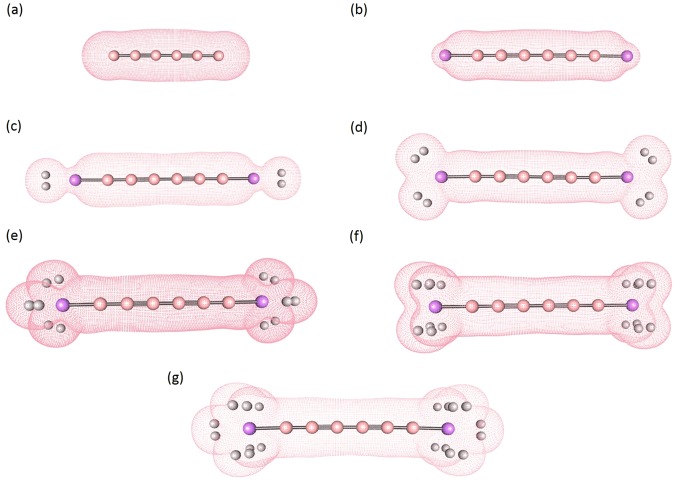
Isosurfaces of charge density (the isovalue is 0.02 e/Å^3^) for (**a**) B_6_ and (**b**–**g**) Li_2_B_6_ with *x* H_2_ molecules (*x* = 0–5) adsorbed on each Li, obtained with TAO-BLYP-D. Here, pink, purple, and white balls represent B, Li, and H atoms, respectively.

To examine the nature of the hydrogen binding energies on Li_2_B_*n*_, the Li atomic charge for Li_2_B_*n*_ (*n* = 6, 8, …, and 16) with *x* H_2_ molecules (*x* = 0–5) adsorbed on each Li (see Fig. 9), is calculated by the CHELPG (CHarges from ELectrostatic Potentials using a Grid based method) scheme^62^. In addition, the isosurfaces of charge density for B_6_ and Li_2_B_6_ with *x* H_2_ molecules (*x* = 0–5) adsorbed on each Li are also plotted (see Fig. 10). For the longer Li_2_B_*n*_, the isosurfaces of charge density remain similar. The charge transfer is from Li to B_*n*_ in Li_2_B_*n*_ due to the difference between their electronegativity values, yielding 0.6–0.7 |*e*| on each Li for Li_2_B_*n*_. This is also evidenced by the depleted charge around each Li. While the charge depleted Li is able to bind more than one H_2_ molecule, the Li atomic charge reduces with the number of the adsorbed H_2_ molecules (*x* = 0–3). This kind of adsorption should be due to that the charge depleted Li can polarize the incoming H_2_ molecules (i.e., governed by the aforementioned charge-induced dipole interactions^2,25,26^), yielding the enhanced hydrogen binding energy and high hydrogen storage capacity for Li_2_B_*n*_. However, when there are many H_2_ molecules adsorbed on each Li (e.g., *x* = 4), the charge densities of the Li atom and the adsorbed H_2_ molecules can be substantially overlapped, which can enhance orbital interactions^3,7^. Therefore, when there are many H_2_ molecules adsorbed on each Li, orbital interactions are expected to be important for the hydrogen binding energies as well. Because of the enhanced orbital interactions, when the fourth H_2_ molecule is adsorbed on the Li atom, some electronic charge can be moved from the Li atom to the adsorbed H_2_ molecules, yielding a slight increase in the positive charge on Li. As the fifth H_2_ molecule is adsorbed very weakly (primarily due to vdW interactions), there is no significant change in the charge on Li. Consequently, the hydrogen adsorption in Li_2_B_*n*_ can be due to not only charge-induced dipole interactions, but also orbital interactions and vdW interactions.

For practical applications, we estimate the desorption temperature, *T*_*D*_, of the adsorbed H_2_ molecules by

9 $${T}_{D}=\frac{{E}_{b}({{\rm{H}}}_{2})}{{k}_{B}}{\{\frac{{\rm{\Delta }}S}{R}-\mathrm{ln}\frac{{p}_{0}}{{p}_{eq}}\}}^{-1}.$$

Note that Eq. (9) is the van’t Hoff equation^55,57,63,64^, where *E*_*b*_(H_2_) is calculated using Eq. (7). As suggested by previous studies^55,57^, the total entropy change before and after the hydrogenation, Δ*S*, is approximated by the change in hydrogen entropy from gas to liquid phase (Δ*S* = 13.819*R*^65^). Besides, *p*_0_, *p*_*eq*_, *k*_*B*_, and *R* are the standard atmospheric pressure (1 bar), the equilibrium pressure, the Boltzmann constant, and the gas constant, respectively. As listed in Table 1, *T*_*D*_ for Li_2_B_*n*_ (*n* = 6, 8, …, and 16) with *x* H_2_ molecules (*x* = 1–4) adsorbed on each Li, is calculated by Eq. (9) at *p*_*eq*_ = 1.5 bar^8^ and at *p*_*eq*_ = 1 bar. Since the *E*_*b*_(H_2_) values range from 21.13 to 26.42 kJ/mol per H_2_ for *x* = 1–4, the respective *T*_*D*_ values range from 179 to 223 K at *p*_*eq*_ = 1.5 bar, and range from 184 to 230 K at *p*_*eq*_ = 1 bar. These desorption temperatures are all well above 77 K (i.e., the boiling point of liquid nitrogen), which can be easily achieved. Therefore, Li_2_B_*n*_ (*n* = 6, 8, …, and 16) can be promising HSMs for storing and releasing H_2_ at temperatures much higher than the boiling point of liquid nitrogen.

**Table 1:** Hydrogen desorption temperature *T*_*D*_ (K) [calculated using Eq. (9) at *p*_*eq*_ = 1.5 (bar) and at *p*_*eq*_ = 1 (bar)] and hydrogen gravimetric storage capacity *C*_*g*_ (wt%) [calculated using Eq. (10)] for Li_2_B_*n*_ (*n* = 6, 8, …, and 16) with *x* H_2_ molecules (*x* = 1–4) adsorbed on each Li, obtained with TAO-BLYP-D.

*n*	*T*_*D*_								*C*_*g*_
	*p*_*eq*_ = 1.5				*p*_*eq*_ = 1				
	1 H_2_	2 H_2_	3 H_2_	4 H_2_	1 H_2_	2 H_2_	3 H_2_	4 H_2_	
6	190	179	186	190	196	184	191	195	17.0
8	198	184	189	191	204	189	195	196	13.8
10	205	188	192	192	211	194	197	197	11.7
12	211	192	194	192	218	198	200	198	10.1
14	218	195	197	194	224	201	202	199	8.9
16	223	199	199	195	230	205	204	200	7.9

Here, *C*_*g*_ is calculated only for *x* = 4.

Since Li_2_B_*n*_ (*n* = 6, 8, …, and 16) is able to adsorb a total of 8 H_2_ molecules (i.e., 4 per Li), where both the average hydrogen binding energies and successive hydrogen binding energies are in the aforementioned desirable regime, we calculate the respective hydrogen gravimetric storage capacity using^57^

10 $${C}_{g}=\frac{8{M}_{{{\rm{H}}}_{2}}}{{M}_{{{\rm{Li}}}_{2}{{\rm{B}}}_{n}}+8{M}_{{{\rm{H}}}_{2}}},$$

where ${M}_{{{\rm{Li}}}_{2}{{\rm{B}}}_{n}}$ is the mass of Li_2_B_*n*_, and ${M}_{{{\rm{H}}}_{2}}$ is the mass of H_2_. As shown in Table 1, *C*_*g*_ ranges from 7.9 to 17.0 wt%, achieving the USDOE ultimate goal of 6.5 wt%. It can be inferred from the H_2_ adsorption patterns of Li_2_B_*n*_ that Li_2_B_*n*_ is able to adsorb up to a total of 8 H_2_ molecules with both the average hydrogen binding energies and successive hydrogen binding energies being in the desirable regime, independent of the value of *n*. Accordingly, the *C*_*g*_ value of Li_2_B_*n*_ should decrease with increasing chain length. Nevertheless, it may not be justified to directly compare the *C*_*g*_ values presented in this work with the USDOE ultimate goal of 6.5 wt%, as the latter is for the entire system of hydrogen storage (which includes the HSM, surrounding container, insulation equipment, and so on)^6^. However, the *C*_*g*_ values of Li_2_B_*n*_ presented in this work are rather high (particularly for the smaller *n*), when compared with the USDOE ultimate goal. Therefore, the entire systems of hydrogen storage via Li_2_B_*n*_ can still be promising HSMs for storing and releasing H_2_ at temperatures much higher than the boiling point of liquid nitrogen.

## Conclusions

In conclusion, because of the recent developments of TAO-DFT, calculations on large systems possessing radical character are now feasible. Accordingly, it is now possible to look for desirable HSMs among multi-reference systems at the nanoscale (i.e., extremely difficult systems for conventional computational approaches). In this work, the electronic properties (e.g., *E*_*b*_(Li), *E*_ST_, IP_*v*_, EA_*v*_, *E*_*g*_, *S*_vN_, and the occupation numbers of active orbitals) and hydrogen storage properties (e.g., *E*_*b*_(H_2_), *E*_*b*,*y*_(H_2_), *T*_*D*_, and *C*_*g*_) of Li_2_B_*n*_ (*n* = 6, 8, …, and 16) have been studied using TAO-DFT. As the ground states of Li_2_B_*n*_ exhibit multi-reference character, KS-DFT with traditional XC functionals may not reliably predict the properties of Li_2_B_*n*_, and calculations based on *ab initio* multi-reference electronic structure methods can be computationally infeasible due to the large electronic systems considered here. Therefore, it is well justified to adopt TAO-DFT in the present study. From our TAO-DFT results, Li_2_B_*n*_ is able to adsorb a total of 8 H_2_ molecules, where both the average hydrogen binding energies and successive hydrogen binding energies are in the desirable regime (between 20 and 40 kJ/mol per H_2_). Hence, the *C*_*g*_ values of Li_2_B_*n*_ range from 7.9 to 17.0 wt%, achieving the USDOE ultimate goal of 6.5 wt%. Accordingly, Li_2_B_*n*_ could be promising HSMs for storing and releasing H_2_ at temperatures much higher than the boiling point of liquid nitrogen, which can be easily achieved.

Because of recent advances in the synthesis of nanomaterials, it may be feasible to practically realize hydrogen storage via Li_2_B_*n*_. For example, Li_2_B_*n*_ can be adopted as building blocks. As proposed by Liu *et al*.^66^, we can connect Li-coated fullerenes with Li_2_B_*n*_, which may be promising HSMs as well. It will then be necessary to comprehensively study the relevant properties of these systems, which can be a possible future study. Furthermore, as the syntheses of Pt-terminated linear carbon chains have been feasible^67^, the syntheses of Li_2_B_*n*_ may be feasible in near future, and are now open to experimentalists.

## Electronic supplementary material

Supplementary Material
