# Supplementary material for: Electronic and Hydrogen Storage Properties of Li-Terminated Linear Boron Chains Studied by TAO-DFT
Source: Sci Rep. 2018 Sep 10;8:13538. doi: 10.1038/s41598-018-31947-9 (PMC6131515; doi:10.1038/s41598-018-31947-9)
Supplement: Supplementary file 1 — Supplementary Material [file 41598_2018_31947_MOESM1_ESM.pdf]

Supplementary Material to: Electronic and Hydrogen Storage Properties of Li-Terminated Linear Boron Chains Studied by TAO-DFT

Sonai Seenithurai[1] and Jeng-Da Chai[1,2,3,*]

[1] *Department of Physics, National Taiwan University, Taipei 10617, Taiwan*

[2] *Center for Theoretical Physics, National Taiwan University, Taipei 10617, Taiwan*

[3] *Center for Quantum Science and Engineering, National Taiwan University, Taipei 10617, Taiwan*

[*] Author to whom correspondence should be addressed. Electronic mail: jdchai@phys.ntu.edu.tw

LIST OF TABLES

S1 Active orbital occupation numbers (HOMO−5, ..., HOMO−1, HOMO, LUMO, LUMO+1, ..., and LUMO+5) for the lowest singlet state of Li_2B_n, obtained with TAO-BLYP-D. For brevity, HOMO is denoted as H, LUMO is denoted as L, and so on. 3

S2 Average hydrogen binding energy (corrected for BSSE) [in kJ/mol per H_2] on Li_2B_n (n = 6, 8, ..., and 16) as a function of the number of H_2 molecules adsorbed on each Li, obtained with TAO-BLYP-D. 4

S3 Average hydrogen binding energy (uncorrected for BSSE) [in kJ/mol per H_2] on Li_2B_n (n = 6, 8, ..., and 16) as a function of the number of H_2 molecules adsorbed on each Li, obtained with TAO-BLYP-D. The BSSE is given in each parenthesis. 4

S4 Binding energy (corrected for BSSE) [in kJ/mol per H_2] of the yth H_2 molecule (y = 1–5) on Li_2B_n (n = 6, 8, ..., and 16), obtained with TAO-BLYP-D. 5

S5 Binding energy (uncorrected for BSSE) [in kJ/mol per H_2] of the yth H_2 molecule (y = 1–5) on Li_2B_n (n = 6, 8, ..., and 16), obtained with TAO-BLYP-D. The BSSE is given in each parenthesis. 5

TABLE S1. Active orbital occupation numbers (HOMO−5, ..., HOMO−1, HOMO, LUMO, LUMO+1, ..., and LUMO+5) for the lowest singlet state of Li_2B_n, obtained with TAO-BLYP-D. For brevity, HOMO is denoted as H, LUMO is denoted as L, and so on.

n	H−5	H−4	H−3	H−2	H−1	H	L	L+1	L+2	L+3	L+4	L+5
6	2.0000	1.9981	1.9967	1.9603	1.9603	1.0377	1.0377	0.0041	0.0041	0.0004	0.0004	0.0000
8	1.9979	1.9976	1.9800	1.9800	1.7445	1.7445	0.2765	0.2765	0.0011	0.0011	0.0002	0.0002
10	1.9971	1.9831	1.9831	1.8940	1.8940	1.0751	1.0751	0.0501	0.0501	0.0003	0.0003	0.0001
12	1.9856	1.9856	1.9445	1.9445	1.6150	1.6150	0.4441	0.4441	0.0135	0.0135	0.0002	0.0002
14	1.9866	1.9626	1.9626	1.8171	1.8171	1.0859	1.0859	0.1462	0.1462	0.0047	0.0047	0.0001
16	1.9716	1.9716	1.8971	1.8971	1.5271	1.5271	0.5671	0.5671	0.0513	0.0513	0.0020	0.0020

TABLE S2. Average hydrogen binding energy (corrected for BSSE) [in kJ/mol per H_2] on Li_2B_n (n = 6, 8, ..., and 16) as a function of the number of H_2 molecules adsorbed on each Li, obtained with TAO-BLYP-D.

n	6	8	10	12	14	16
1 H_2	22.51	23.40	24.21	24.99	25.72	26.42
2 H_2	21.13	21.76	22.25	22.75	23.09	23.54
3 H_2	21.96	22.36	22.68	22.97	23.24	23.49
4 H_2	22.45	22.54	22.65	22.75	22.89	23.00
5 H_2	18.47	18.68	18.88	19.05	19.20	19.35

TABLE S3. Average hydrogen binding energy (uncorrected for BSSE) [in kJ/mol per H_2] on Li_2B_n (n = 6, 8, ..., and 16) as a function of the number of H_2 molecules adsorbed on each Li, obtained with TAO-BLYP-D. The BSSE is given in each parenthesis.

n	6	8	10	12	14	16
1 H_2	25.16 (2.65)	26.06 (2.65)	26.88 (2.67)	27.67 (2.68)	28.41 (2.69)	29.11 (2.69)
2 H_2	23.67 (2.54)	24.27 (2.51)	24.75 (2.51)	25.25 (2.51)	25.58 (2.49)	26.02 (2.48)
3 H_2	24.40 (2.44)	24.79 (2.43)	25.11 (2.43)	25.40 (2.43)	25.65 (2.41)	25.90 (2.41)
4 H_2	24.68 (2.23)	24.73 (2.19)	24.83 (2.18)	24.92 (2.17)	25.05 (2.16)	25.16 (2.16)
5 H_2	20.87 (2.40)	21.05 (2.37)	21.24 (2.37)	21.41 (2.36)	21.56 (2.36)	21.71 (2.36)

TABLE S4. Binding energy (corrected for BSSE) [in kJ/mol per H_2] of the yth H_2 molecule (y = 1–5) on Li_2B_n (n = 6, 8, ..., and 16), obtained with TAO-BLYP-D.

n	6	8	10	12	14	16
$1^{st}H_2$	22.51	23.40	24.21	24.99	25.72	26.42
$2^{nd}H_2$	19.76	20.12	20.28	20.50	20.46	20.67
$3^{rd}H_2$	23.61	23.57	23.55	23.42	23.54	23.39
$4^{th}H_2$	23.91	23.08	22.57	22.08	21.84	21.52
$5^{th}H_2$	2.54	3.25	3.77	4.25	4.46	4.76

TABLE S5. Binding energy (uncorrected for BSSE) [in kJ/mol per H_2] of the yth H_2 molecule (y = 1–5) on Li_2B_n (n = 6, 8, ..., and 16), obtained with TAO-BLYP-D. The BSSE is given in each parenthesis.

n	6	8	10	12	14	16
$1^{st}H_2$	25.16 (2.65)	26.06 (2.65)	26.88 (2.67)	27.67 (2.68)	28.41 (2.69)	29.11 (2.69)
$2^{nd}H_2$	22.18 (2.43)	22.48 (2.36)	22.62 (2.34)	22.83 (2.33)	22.75 (2.29)	22.93 (2.27)
$3^{rd}H_2$	25.87 (2.25)	25.83 (2.27)	25.84 (2.29)	25.69 (2.27)	25.78 (2.24)	25.66 (2.27)
$4^{th}H_2$	25.51 (1.60)	24.56 (1.48)	23.98 (1.41)	23.47 (1.39)	23.25 (1.40)	22.92 (1.40)
$5^{th}H_2$	5.61 (3.07)	6.33 (3.08)	6.88 (3.11)	7.38 (3.13)	7.63 (3.17)	7.94 (3.18)
